# Supplementary material for: “Notame”: Workflow for Non-Targeted LC–MS Metabolic Profiling
Source: Metabolites. 2020 Mar 31;10(4):135. doi: 10.3390/metabo10040135 (PMC7240970; doi:10.3390/metabo10040135)
Supplement: Supplementary file 1 [file metabolites-10-00135-s001.pdf]

metabolites

Supplementary Material

"notame": Workflow for Non-Targeted LC–MS Metabolic Profiling

Anton Klåvus [1,†,*], Marietta Kokla [1,†,*], Stefania Noerman [1], Ville M. Koistinen [1], Marjo Tuomainen [1], Iman Zarei [1], Topi Meuronen [1], Merja R. Häkkinen [2], Soile Rummukainen [2], Ambrin Farizah Babu [1], Taisa Sallinen [1,2], Olli Kärkkäinen [2], Jussi Paananen [3], David Broadhurst [4], Carl Brunius [5,6] and Kati Hanhineva [1, 5, 7*]

[1] University of Eastern Finland, Department of Clinical Nutrition and Public Health, 70210 Kuopio, Finland; stefania.noerman@uef.fi (S.N.); ville.m.koistinen@uef.fi (V.M.K.); marjo.tuomainen@uef.fi (M.T.); iman.zarei@uef.fi (I.Z.); topi.meuronen@uef.fi (T.M.); ambbab@student.uef.fi (A.F.B.); taisa.sallinen@uef.fi (T.S.)
[2] University of Eastern Finland, School of Pharmacy, 70210 Kuopio, Finland; merja.hakkinen@uef.fi (M.R.H.); soile.rummukainen@uef.fi (S.R.); olli.karkkainen@uef.fi (O.K.)
[3] University of Eastern Finland, Institute of Biomedicine, 70210 Kuopio, Finland; jussi.paananen@uef.fi
[4] Centre for Integrative Metabolomics & Computational Biology, School of Science, Edith Cowan University, Joondalup 6027, Australia; d.broadhurst@ecu.edu.au
[5] Chalmers University of Technology, Department of Biology and Biological Engineering; Gothenburg 41296, Sweden; carl.brunius@chalmers.se
[6] Chalmers University of Technology, Chalmers Mass Spectrometry Infrastructure, Gothenburg 41296, Sweden
[7] University of Turku, Department of Biochemistry, Food Chemistry and Food Development unit, 20014 Turun yliopisto, Finland
† These authors have contributed equally to this work.
* Correspondence: anton.klavus@uef.fi (A.K.); marietta.kokla@uef.fi (M.K.); kati.hanhineva@uef.fi (K.H.)

Section 1. Clustering of features originating from the same compound

Features originating from the same compound are assumed to be strongly correlated and have a small difference in their retention time. This motivates the first step of the algorithm: the algorithm identifies pairs of correlated features within a specified retention time window. The user specifies both the correlation threshold and the size of the retention time window. For illustration, a correlation coefficient threshold of 0.9 and a retention time window of ±1 second is used. Pearson correlation coefficient is used, as the relationship between features originating from the same compound is assumed linear.

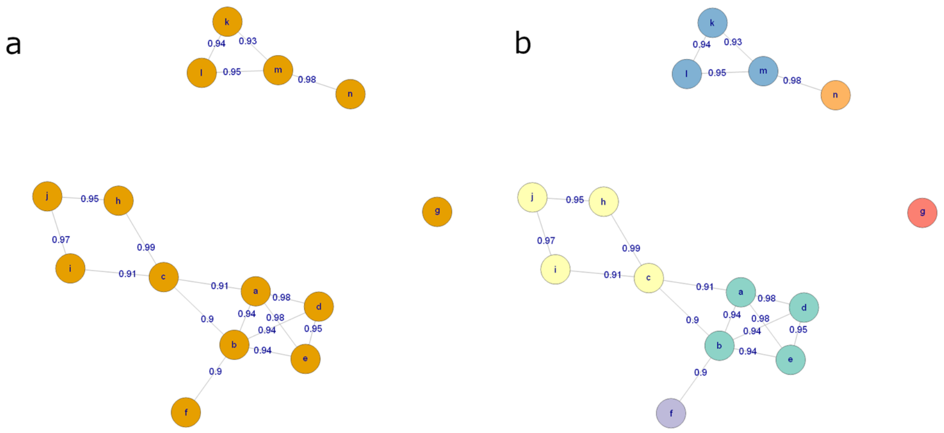

Figure S1 (Same as Figure 12 of the main text). a) An example graph, where every node is a molecular feature and every edge represents a high correlation coefficient and a small retention time difference between the features. b) The graph after the clustering procedure. Each color corresponds to a distinct cluster of features.

Next, an undirected graph of all the connections between features is generated; where each node represents a feature, each edge an aforementioned connection and edge weight the corresponding Pearson correlation coefficient, see Figure S1 for an example. The graph is then decomposed to its connected components, groups of nodes where all the nodes of the component are reachable from any other node. These components can contain features that are connected to only one other feature out of many. Removing these features will reduce the number of false annotations. Thus, the next step is to prune the components.

Nodes are removed from the component until all the nodes have a sufficiently high degree (the number of edges of the node). This step requires a third user-defined parameter, degree threshold, defined as a percentage of the maximum possible degree. For example, in a component of five nodes, the maximum degree is 4. With a degree threshold of 0.8, each node is required to have at least $0.8 \cdot 4 = 3.2 \approx 3$ edges (the number of edges required is rounded to the nearest integer). If this criterion is not met, the node with the lowest degree is discarded until the criterion is met. In the case of a tie, the node with the lowest sum of edge weights is discarded. Note that nodes that are initially discarded can form new clusters. Figure S2 illustrates the process of the algorithm on the largest component of the graph in Figure S1. After the clustering, the feature with the largest median peak area is retained for each cluster. All the features that are clustered together are recorded for future reference.

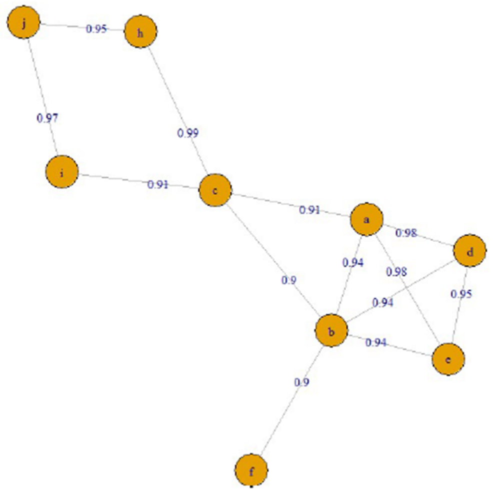

(a) The original cluster, clearly node f will be dropped.

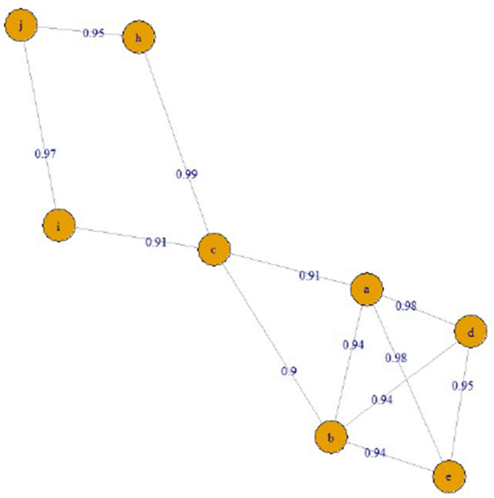

(b) Nodes i, j and h all have degree 2. Since node i has the lowest sum of edge weights (correlations), it will get dropped next.

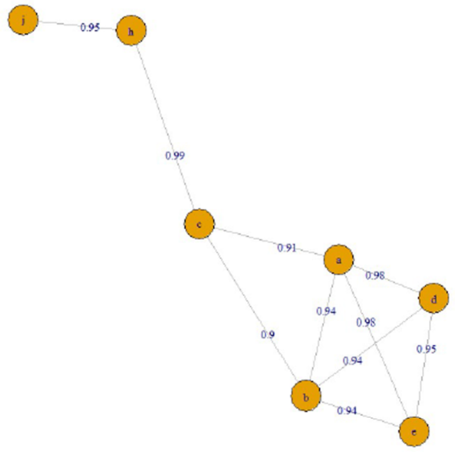

(c) Nodes j and h are the next nodes to be dropped.

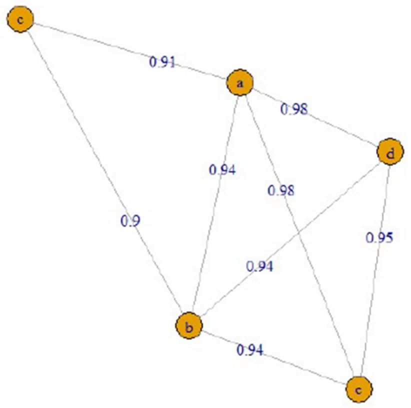

(d) Node c will get dropped next, as it has the lowest degree.

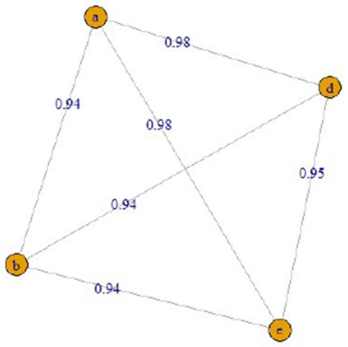

(e) Finally, each node is connected to all other nodes, so the cluster is finished.

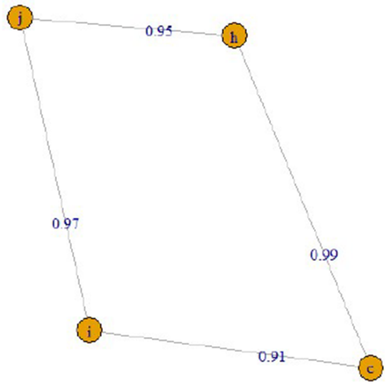

(f) The dropped nodes will form another cluster, since the required threshold for the degree $0.8 \cdot 3 = 2.4 \approx 2$ is fulfilled for each node.

Figure S2. Description of the pruning process for clusters in the graph depicted in Figure S1.

Section 2. Tutorial on Pathway Analyses Tools

MetaboAnalyst (https://www.metaboanalyst.ca/)

a. Enrichment Analysis
    i. Paste the list of the HMDB or KEGG ID to the list of a compound names for the over-representation analysis in the Enrichment Analysis module of MetaboAnalyst 4.0
    ii. Specify the ID type, then click "Submit"
    iii. Choose "pathway-associated metabolite sets (SMPDB)" and use only metabolites sets containing at least 2 compounds
    iv. Upload a reference metabolome based on your analytical platform or use all compounds in the selected metabolite set library, then click "Submit"
    v. Note that the results would be available both in network and bar view, then click "Submit" to be redirected to the download links.
    vi. Download download.zip to download all results files, or select only certain files you need.
b. Pathway Analysis
    vii. Paste the list of the HMDB or KEGG ID in the Pathway Analysis module in MetaboAnalyst 4.0
    viii. Choose *Homo sapiens* KEGG as the reference library for human-based samples, or other libraries depending on sample origin
    ix. Depending on what you want, choose the test for over-representation analysis and pathway topology. We use Fischer exact test for and relative-betweenness centrality, respectively
    x. Upload your reference metabolome, or use all compounds in the selected pathway
    xi. Import the results
c. Network Explorer
    xii. Paste the list of the HMDB or KEGG ID in the Network Explorer module in MetaboAnalyst 4.0
    xiii. Check the list of the metabolites, delete the unrecognized ones
    xiv. Choose "metabolite-metabolite interaction network" mode if you only upload the list of metabolites.
    xv. Click "Proceed" to view the network
    xvi. Click "Download" to import the results

MetScape (http://metscape.ncibi.org/).

Build a network:

a. Open the Cytoscape software.
b. Choose the "Apps" menu on the tool bar.
c. Choose the first option "App Manager" from the dropdown menu.
d. In App Manager window, in the search box, enter "MetScape". MetScape should appear in the second column.

e. Click on “MetScape” and then Click on “Install” at the bottom of the window. Once the app is installed, it will appear in the “Apps” menu. One-time free registration is required the first time, the MatScape is opened.
f. From “Apps” menu, “MetScape”, click the “Built Network” and then “Pathway-based”.
g. In “Input” section, choose your Organism (Human, Plant, or Mouse).
h. In “Import” section, “Select” button upload the experimental data (*.CSV). The MetScape main window has three tabs that provide users with the following options Load a list of compounds, one compound per line (compound names, KEGG IDs, HMDB IDs, BIGG IDs, EHMN IDs), or load a file containing normalized experimental metabolite data with metabolite KEGG IDs and corresponding values at given time points or under specific experimental conditions.
i. Under “Options” section, “Network Type”, Choose pathway-specific networks by choosing a pathway from the drop-down list.
j. click “Build Network” at the bottom of Control Panel.

Correlation Calculator (optional step)

Correlations are measures between pairs of metabolites. Correlation Calculator is a standalone Java application that provides methods of calculation pairwise correlations among repeatedly measured entities. It is designed for use with quantitative metabolite measurements, such as Mass Spectrometry data, on a set of samples. The workflow allows inspection and/or saving of results at various stage, and the final correlation results can be dynamically imported into version 3.1 or higher of MerScape as a correlation network.

a. The Correlation Calculator can be downloaded from MetScape website (http://metscape.ncibi.org/CorrelationCalculator-1.0.1.jar).
b. The input data file is a CSV file that contains a table of measurements across multiple samples. Although metabolites must be labeled, sample labels are optional.
c. Samples may be in rows or columns.
d. After launching the calculator, click the browse button.
e. Select the appropriate data file and click Open (make sure to specify the file format.
f. Under, “Data Normalization”, Select “Log2-Transform Data” AND “Autoscale Data” and click “Run”.
g. Click “View Normalized Data” to view the results. To save the data click the “Save” button. if the data are already normalized before loading it into the calculator, this normalized step can be skipped.
h. Pearson’s Correlations is performed to filter out metabolites; this step is optional. To use Pearson’s Correlations, click “Run” under “Data Alalysis”. Histogram and heatmap view are available for this analysis.
i. The last step is to use a Partial Correlation Method, either Debiased Sparse Partial Correlation (DSPC) or Basic Partial Correlation, and then click “Run”. The Correlation Calculator calculates the partial correlation values, *p*-values, and *q*-values for each compound pair.
j. To view the result in the MetScape, click “View in MetScape” where interactive visualization and exploration can be performed.

Correlation network:

To build a correlation network in MetScape, appropriate data file formatting is required. Two types of data file formats are accepted. The first data file format is column-based (recommended format). The first row of column-based file must have column heading of the user’s choosing. The first two columns must contain metabolite names or IDs. Additional columns contain values such as *p*-values, *q*-values, and correlation values. The second data file format is a matrix format, where the first row and the first column contain metabolite names, and the rest of the rows and columns contain correlation values.

a. Open the Cytoscape
b. Go to the "Apps" menu and click on "MetScape".
c. Select "Build Network" and then "Correlation-based". Now, a MetScape tap displays on the left side of the screen, in the Control Panel.
d. Under the "Input" section, click the "Select" button. Select the location of the correlation file and click open.
e. A new window will appear showing potential matches found in the MetScape database, for each compound in the input file.
f. Use the dropdown arrows for each compound to choose the best match. If the compound is not found in the system, it will say "Not Found". Your mapping selection will be saves, so that it will appear as the default option in the future.
g. Select "OK".
h. Under "Edge Mapping" in Control Panel, use the dropdown menu next to "Base Edges on" and select the appropriate column from your data file (e.g. correlation values column.
i. Under "Edge Mapping" in Control Panel, use the dropdown menu next to "Tooltip Labels" and select the appropriate column from your data file (e.g. *p*-values column).
j. Click "Build Network".
